# Widefield Swept Source OCTA in Retinitis Pigmentosa

**DOI:** 10.3390/diagnostics10010050

**Published:** 2020-01-19

**Authors:** Rodolfo Mastropasqua, Rossella D’Aloisio, Chiara De Nicola, Giada Ferro, Alfonso Senatore, Daniele Libertini, Guido Di Marzio, Marta Di Nicola, Giuseppe Di Martino, Luca Di Antonio, Lisa Toto

**Affiliations:** 1 Institute of Ophthalmology, University of Modena and Reggio Emilia, Via Università, 4, 41121 Modena (MO), Italy; rodolfo.mastropasqua@gmail.com; 2 Ophthalmology Clinic, Department of Medicine and Science of Ageing, University G. d’Annunzio Chieti-Pescara, 66100 Chieti, Italy; chiaradenicola@gmail.com (C.D.N.); giadaferro90@hotmail.it (G.F.); doctor_alf@hotmail.it (A.S.); daniele.libertini@hotmail.com (D.L.); dimarzio61@alice.it (G.D.M.); monsieurluca@yahoo.com (L.D.A.); l.toto@unich.it (L.T.); 3 Duke University Eye Center, Center for Retinal Degenerations and Ophthalmic Genetic Diseases, Durham, NC 27705, USA; 4 Laboratory of Biostatistics, Department of Medical, Oral and Biotechnological Sciences, University “G. d’Annunzio” Chieti-Pescara, via dei Vestini 31, 66100 Chieti, Italy; mdinicola@unich.it; 5 Department of Medicine and Science of Ageing, School of Hygiene and Preventive Medicine, University G. D’Annunzio Chieti-Pescara, via dei Vestini 31, 66100 Chieti, Italy; peppinodimartino@hotmail.com

**Keywords:** retinitis pigmentosa, widefield swept source optical coherence tomography angiography, retinal perfusion density

## Abstract

(1) Background: To evaluate superficial capillary plexus (SCP), deep capillary plexus (DCP), choriocapillaris (CC), perfusion density (PD), and vessel length density (VLD) in macular and near/mid periphery regions in patients with retinitis pigmentosa (RP) using widefield swept source optical coherence tomography angiography (WSS-OCTA). (2) Methods: Twelve RP patients (20 eyes) and 20 age-matched subjects (20 eyes) were imaged with the SS-OCTA system (PLEX Elite 9000, Carl Zeiss Meditec Inc., Dublin, CA, USA). Quantitative analysis was performed in the macular and peripheral regions. The main outcome measures were SCP, DCP, CC, PD, and VLD in central and peripheral areas. (3) Results: Mean visual acuity, central macular thickness, and microperimetry were significantly reduced in RP patients compared to normal subjects (*p* < 0.05). The perfusion density and VLD of SCP, DCP, and CC were significantly reduced in RP patients compared to normal controls both in the central and peripheral retina (*p* < 0.05). A significant direct correlation was found in RP patients between PD of the 1.5 mm central retina both in DCP and CC and microperimetry at 4° and 8°. (4) Conclusions: Widefield SS-OCTA shows an impairment of retinal and choroidal perfusion density and vessel length density in central and peripheral retina of RP patients. The reduction of flow features correlates with the macular function.

## 1. Introduction

The relationship between ocular vascularization and retinitis pigmentosa (RP) has not been fully understood. Changes of retinal vasculature with vessel attenuation observed by fundoscopy are a hallmark of the disease [1].

Histopathological studies also showed that the reduced ocular blood flow is a primary event due to retinal vessel damage with vessel narrowing, followed by thickening of the blood vessel wall, that lead to lumen occlusion [2,3].

Retinal blood flow velocities evaluated using retinal function imaging techniques have also been found to be lower in patients with RP compared to healthy subjects [4].

Changes in retinal and choroidal vessels have been recently described in RP patients by means of optical coherence tomography angiography (OCTA), confirming previous clinical and histological data. In particular, flow features alterations of both choroidal and retinal vessels have been described correlating with macular function impairment [5,6,7].

The recent introduction of widefield OCTA with longer wavelengths and higher speed allows better analysis of deeper tissue such as choriocapillaris and the visualization of a wider retinal field of view. In this way, it provides more details of retinal and vascular disorders not limited to the posterior pole [8].

In RP, the primary defect lies in the rod photoreceptors thus beginning in the far and midperipheral retina, later involving the cone photoreceptors localized more centrally.

Widefield OCTA technology is being used to explore retinal vascular disorders from the macular region to the periphery with the disadvantage of artifacts complicating widefield OCTA imaging [9,10,11,12].

The aim of this study is to assess retinal vascular plexuses and choriocapillaris features in selected retinal areas from the foveal zone toward mid-peripheral retina in RP patients using widefield swept source OCTA (WSS-OCTA) compared to the vascular features of healthy subjects and to establish the macular function of these patients using microperimetry and electrophysiological testing.

## 2. Materials and Methods

### 2.1. Study Participants

In this prospective observational study, 12 patients with previous diagnosis of either mid- or late-stage RP and a control group of 20 healthy frequency age-matched subjects were referred to the University G. d’Annunzio, Chieti-Pescara, Italy.

This prospective study was approved by the Institutional Review Board and adhered to the tenets of the Declaration of Helsinki. All participants gave their written informed consent. Criteria for inclusion for RP group were: (1) age > 18 years old; (2) best-corrected visual acuity (BCVA) greater than 0.9 logMAR in the study eye at baseline examination (to ensure proper execution of examination); (3) clinical, genetic and electrophysiological diagnosis of retinitis pigmentosa.

The inclusion criteria for controls were: (1) age >18 years old; (2) spherical refraction ≤ 3.0 D and cylinder correction ≤ 2.0 D; normal ophthalmoscopic appearance of retinal fundus.

The exclusion criteria of RP and healthy eyes were: (1) any ocular surgery (included intravitreal injections) in the study eye in the last 9 months; (2) laser treatment in the study eye; (3) history of glaucoma; (4) media opacity in the study eye; (5) any other ocular and retinal disease; (6) ocular axial length not included between 23 and 24 mm.

### 2.2. Procedures

All patients recruited between September 2018 and February 2019 underwent a complete ophthalmologic examination, including BCVA evaluation using Early Treatment Diabetic Retinopathy Study (ETDRS) charts, tonometry, slit-lamp biomicroscopy and indirect fundus ophthalmoscopy. In addition, all patients underwent full field electroretinogram (ERG) and multifocal electroretinogram (mfERG) with the Retimax (CSO, Scandicci, Florence, Italy), microperimetry (MP) by means of a MP-3 Microperimeter (Nidek Technologies, Padova, Italy), spectral domain OCT (SD OCT) (Spectralis, HRA Heidelberg, Heidelberg, Germany) and OCTA using PLEX Elite 9000 device (Carl Zeiss Meditec Inc., Dublin, CA, USA).

### 2.3. Electrophisiology Testing

Full-field electroretinography and mfERGs were recorded for each patient according to the International Society for Clinical Electrophysiology of Vision (ISCEV) protocols [13,14].

The amplitudes and peak times of b waves of dark-adapted 0.01 ERG, dark-adapted 3.0 ERG and light-adapted 3.0 ERG and the amplitude of the light-adapted 30 Hz flicker ERG were measured for all patients.

In the mfERG, the ocular fundus was segmented by an array of 61 hexagons testing a central retinal area of 25°, and average responses for the implicit times and amplitudes of N1 (first negative component) and P1 (first positive component) of the first-order kernel were calculated for five regional ring groups (R1 to R5). Amplitude was measured from the baseline to the trough (N1) or the peak (P1) of the deflection. N1 to P1 amplitudes (from the first negative to the first positive peak) on MfERGs in the five recorded rings was considered for analysis.

Patients were instructed to look at a fixation point incorporated into the stimulus dome for ERG or to the centre of a cross on a video display for MfERGs to ensure accurate execution of the exam. Correct fixation was monitored with the help of an infrared CCD camera positioned in the ganzfeld stimulator during ERG or by direct observation of the patient fixating on the video display during MfERG acquisition.

### 2.4. Microperimetry

Microperimetry was performed by means of the MP-3 Microperimeter. All patients were dilated with tropicamide 1% eye drops, and after pre-test training, 5 min was allotted for adaptation to the dark. This test is routinely carried out with an automated eye tracking system as previously described [5]. To assess central macular retinal sensitivity, differential light threshold values were compared by calculating the mean of the 4°, 8°, and 20° of the macular area, which was averaged automatically by the MP-3 software programme for the mean sensitivity in a polygon.

To assess fixation, fundus movements were tracked during the examination as previously described [5]. To improve the correlations between microperimetric data with retinal characteristics, the results were matched with a colour digital retinography obtained with an MP-3 colour fundus camera.

### 2.5. SD-OCT

The acquisition protocol for SD OCT (Spectralis, HRA Heidelberg, Heidelberg, Germany) included a 49 horizontal raster dense linear B-scans centered on the fovea. A horizontal and vertical B-scans centered on the fovea were also acquired in all patients.

Central macular thickness was measured using the central 1-mm-diameter circle of the ETDRS thickness map.

### 2.6. OCTA

Patients underwent OCTA imaging using the PLEX Elite 9000 device (Carl Zeiss Meditec Inc., Dublin, CA, USA), which uses a swept laser source with a central wavelength of 1050 nm (1000–1100 nm full bandwidth) and operates at 100,000 A-scans per second. This instrument employs a full-width at half-maximum (FWHM) axial resolution of approximately 5 μm in tissue, and a lateral resolution at the retinal surface estimated at approximately 14 μm [8].

For each eye, a 12 × 12 mm OCTA volume scan was acquired. FastTrack motion correction software was used while the images were acquired. Poor quality images (signal strength index (SSI) < 8) with either significant motion artifact or extensive incorrect segmentation were excluded and repeated.

**Figure 1:**
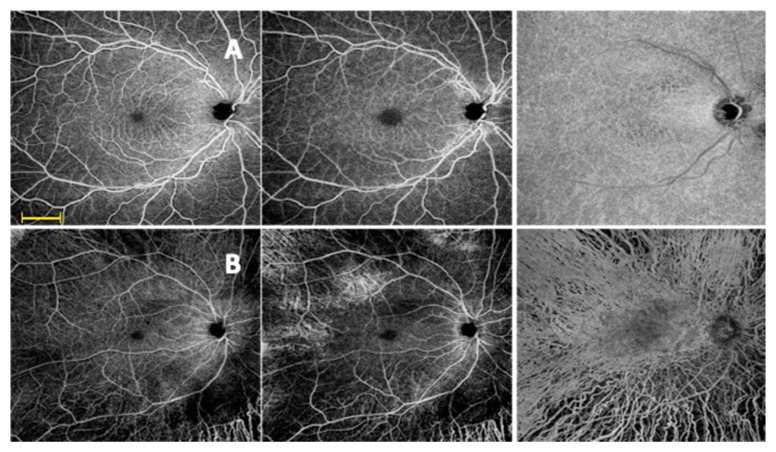
Widefield swept source optical coherence tomography (WSS-OCTA) assessment of superficial capillary plexus (left images), deep capillary plexus (middle images) and choriocapillaris (right images) in a 12 × 12 mm image of healthy eye (**A**) and retinitis pigmentosa (RP) patient (**B**). Scale bar = 3 mm.

For all the participants, both eyes were imaged separately 3 times each, and the best quality image from each eye was selected to be analyzed in the study (Figure 1).

All selected images were carefully visualized by two retinal specialist independently (CDN and LT) to ascertain the correctness of the position of the upper and lower boundaries of segmentation such as the inner limiting membrane (ILM) and retinal pigment epithelium (RPE) respectively and in case of erroneous boundaries recognition manual correction was performed using the segmentation and propagation editing tool from the device. Then automatic segmentation by PLEX Elite 9000 device was used to define vascular beds obtaining 3 depth resolved retinal slabs: the superficial capillary plexus extends from the internal limiting membrane (ILM) to the inner plexiform layer (IPL), the deep capillary plexus extends from the IPL to the outer plexiform layer (OPL) and the choriocapillaris consisting of a 20 μm thick uniform layer extending 29 μm below the RPE to 49 μm below the RPE.

The main outcome measures were perfusion density (PD) and vessel length density (VLD) of superficial capillary plexus (SCP), deep capillary plexus (DCP) and choriocapillaris (CC).

The details of the methods used to quantify these variables have been previously described [15,16].

For each eye, en face OCTA images segmented at the SCP, DCP, and CC levels were imported into ImageJ software version 1.50 (National Institutes of Health, Bethesda, MD; available at http://rsb.info.nih.gov/ij/index.html).

Each SCP and DCP en face image were duplicated and binarized with two different binarization methods (“Huang’s fuzzy” method and “median local” thresholding) to calculate perfusion density, as previously described.

To evaluate CC perfusion density, en face images were binarised using the Phansalkar method and then processed with the ‘analyze particles’ command as previously shown.

The DCP and CC directly beneath major superficial retinal vessels were excluded from analysis to eliminate potentially confounding shadow or projection artifacts as previously described [17].

**Figure 2:**
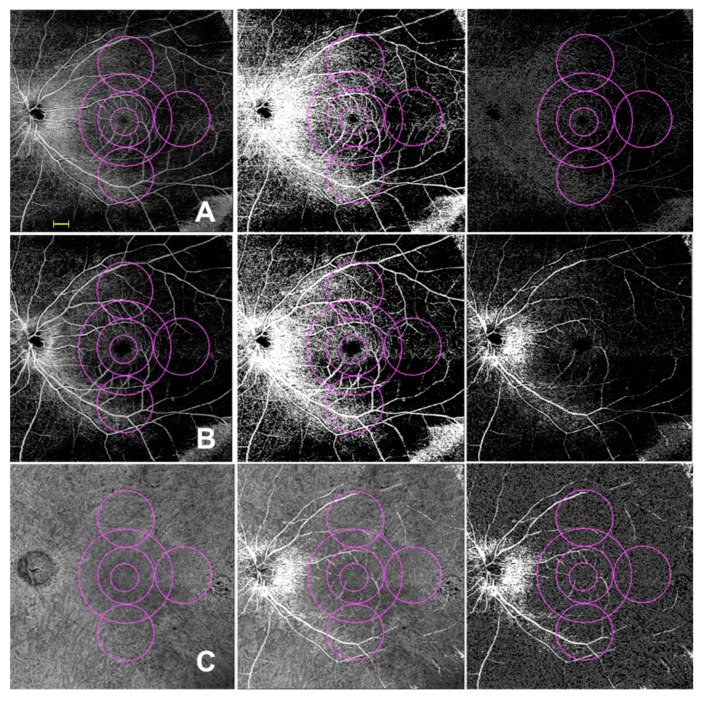
Widefield swept source optical coherence tomography (WSS-OCTA) assessment of superficial capillary plexus (SCP) (**A**). deep capillary plexus (DCP) (**B**) and choriocapillaris (CC) (**C**) in a 12 × 12 mm image of a retinitis pigmentosa (RP) patient; **A**–**C** (Left images) SCP, DCP and CC were investigated in two different regions: macular region (three concentric pink circles with a diameter of 1.5 mm. 3 mm ad 5 mm); and midperiphery region (three pink circles with diameters of 3 mm tangential to the central circle). The SCP, DCP and CC binarized (**A**–**C** middle) and skeletonized (**A**–**C** right) images were obtained with ImageJ to investigate perfusion and vessel length density, respectively. The superficial capillary plexus’ big retinal vessels were identified and merged into DCP and CC images and finally excluded from the analysis, thus avoiding shadowing and projection artifacts from SCP. Scale bar = 1 mm.

Successively, the SCP, DCP, and CC images obtained after binarization were skeletonized and these images were employed to measure VLD (Figure 2).

Quantitative analysis was performed in the macular region, which was defined as three concentrical circular annulus around the fovea with diameter of 1.5 mm, 3 and 5 mm (respectively called “tight central ring”, “medium central ring” and “large central ring”) and in the peripheral region which was assessed in three circles tangential (“superior ring”, “temporal ring” and “inferior ring”) to the macula and with diameters of 3 mm (Figure 2).

### 2.7. Statistical Analysis

Assuming an expected difference in tight central choroidal vessel length density between cases and controls of 18%, with a pooled standard deviation of 17, a power level of 80% and an alpha error of 5%, this study required at least 20 eyes for each study group.

Categorical variables were expressed as frequency and percentage. Continuous variables were expressed as mean and standard deviation (SD) or median and interquartile range (IQR) according to their distribution. Shapiro-Wilk’s test was performed to evaluate the departure from normal distribution of continuous variables. Student’s t-test for independent samples or Mann-Whitney U-test were performed to evaluate differences in baseline characteristics between cases and controls group. Mean differences in patient characteristics were calculated as controls minus cases values.

Different linear models were applied to evaluate the effect of age on the evaluated parameters.

Pearson’s correlation coefficient was estimated to evaluate the correlation among central and peripheral sectors of vessel density and length of vessel, according to study group. Correlation coefficient was also estimated to evaluate correlation among central sectors of vessel and length of vessel with functional parameters.

All statistical analyses were performed using R Statistical Software (version 3.6.1; R Foundation for Statistical Computing, Vienna, Austria). All tests were two-tailed, and a *p*-value < 0.05 was considered indicative of a statistically significant association.

## 3. Results

### 3.1. Demographics of Subjects Included in the Analysis

Twenty eyes of the 12 RP patients included in the study met the required image quality criteria and were used in the analysis. Twenty eyes of 20 healthy subjects were included as controls. Mean age was 46.4 ± 15.4 years in the RP group and 45.3 ± 10.7 years in the healthy control group (*p* = 0.796). The range of age in control group was from 23 years to 58 years. The female/male ratio was 6/6 in the in the RP group and 11/9 in the healthy control group (*p* = 0.927). Clinical and genetical features of the RP patients are reported in Table 1.

### 3.2. Visual Acuity, Electrophysiology and Microperimetry

Mean visual acuity was significantly reduced in RP patients compared to normal subjects (*p* = 0.020) (Table 2).

Mean electroretinogram (ERG) and multifocal electroretinogram (mfERG) values of the enrolled RP patients and the healthy subjects are reported in Table 1. ERG amplitudes were significantly reduced and latencies were significantly delayed in the RP group compared to normal subjects (*p* < 0.001). Amplitudes of MfERG were significantly reduced in all of the five rings in RP patients compared to normal subjects (Table 2). A statistically significant reduction of retinal sensitivity was observed in RP patients compared to normal subjects at 4°, 8° and 20° (*p* < 0.05) (Table 3).

### 3.3. SD OCT and Widefield OCTA Analysis of the Retinal Vessels

#### 3.3.1. Central Macular Thickness

Mean central macular thickness (CMT) was 207.68 ± 51.63 µm in RP patients and 252.74 ± 20.03 µm in normal subjects (*p* < 0.001).

#### 3.3.2. Perfusion Density

A significant reduction of perfusion density (PD) was found in five of the six fields considered (large central ring. superior ring. temporal ring. inferior ring and tight central ring) of the superficial capillary plexus (SCP) (*p* < 0.05) compared to normal subjects. Five of the six fields (medium central ring, large central ring, superior ring, temporal ring and inferior ring) of the deep capillary plexus (DCP) (*p* < 0.001) compared to normal subjects and all of the six fields of choriocapillaris (CC) (*p* < 0.001) compared to normal subjects (Table 4).

#### 3.3.3. Vessel Length Density

Vessel length density (VLD) is significantly lower in the large central. in the superior and the inferior rings of the SCP (*p* < 0.001) compared to normal subjects. in all the rings of the DCP (*p* < 0.05) and choriocapillaris (*p* < 0.001) compared to normal subjects. as shown in Table 5.

The interaction term between age and group resulted not statistically significant for all parameters evaluated.

### 3.4. Correlations

A significant direct correlation was found between central and peripheral sectors in RP patients in superior and temporal fields of the SCP (*p* < 0.01) and in the temporal field of the DCP (*p* < 0.001) for vessel length density values as described in Table 6.

A significant positive correlation was found in controls between central and peripheral sectors in all retinal plexuses and choriocapillaris both for PD and VLD. A significant positive correlation was found in RP patients between PD of the tight and medium central rings both in DCP and CC and microperimetry at 4° and 8° (Table 7).

**Figure 3:**
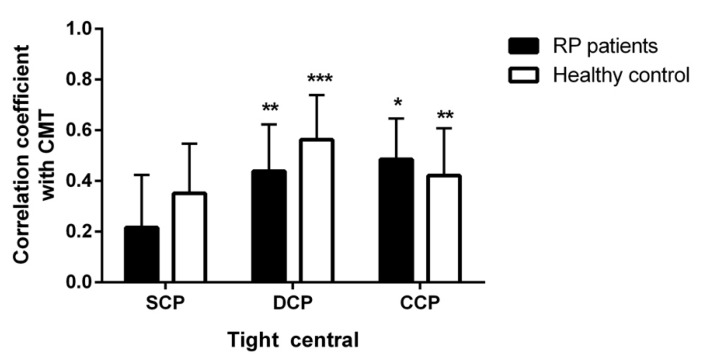
Pearson’s correlation coefficients between PD of the tight central ring in SCP, DCP and CC and CMT in RP patients and healthy controls. Error bar represent standard error of coefficient. * *p* < 0.05; ** *p* < 0.001 and *** *p* < 0.001.

In addition, a significant positive correlation was found in RP patients between PD of the tight central ring in DCP and CC in cases both and CMT (R = 0.439; *p* = 0.001 and R = 0.485; *p* = 0.031) and controls (R = 0.563; *p* < 0.001; and R = 0.421; *p* = 0.002) (Figure 3).

## 4. Discussion

In this prospective observational study, we investigated retinal and choriocapillaris vascular features in selected retinal areas from the foveal zone toward retinal periphery in RP patients using SS-widefield OCT angiography compared to vascular features of healthy subjects and we established macular function of these patients using microperimetry and electrophysiological testing. In general, we found that RP patients were characterized by both central and peripheral retinal and choroidal vessel impairment compared to healthy controls. In addition, microperimetry values were significantly related to perfusion density values.

Alterations in the ocular blood flow and retinal vascularization has been fully described as being a clinical feature of RP and the role of retinal vascular alterations in RP pathogenesis has been hypothesized [1,2,3,4,18].

In recent years, several authors have confirmed choroidal and retinal vessels alterations in RP patients using small field OCTA [5,6,7,19].

Toto et al. showed a significative reduction of the retinal and choriocapillaris vessel densities in RP patients after comparison with healthy subjects. These vascular changes were related to the macular function, as well as to ganglionar cell complex (GCC) thickness [5].

Battaglia Parodi et al. reported a reduction of vessel density and vessel tortuosity and an increase of vessel dispersion and vessel rarefaction with higher visual acuity in patients with worst vascular parameters [6].

Jauregui et al. observed a reduction of perfusion density in the SCP and DCP and an increase of foveal avascular zone area in RP patients during follow up and suggested these parameters as useful markers to monitor disease progression [7].

To the best of our knowledge, there are no widefield OCTA studies assessing retinal vascular and choroidal alterations in RP patients evaluating both the central and peripheral retina.

We demonstrated that PD and VLD are reduced in SCP, DCP and choriocapillaris in patients affected by RP, considering six rings on a 12 × 12 mm widefield OCTA image, being three rings of different size centered on the fovea and the others placed superiorly, temporarily, and inferiorly to the fovea. A significant reduction of PD and VLD was found both in central and peripheral retina of RP patients compared to healthy subjects with greater impairment in DCP and CC.

The difference between RP patients and normal subjects was not statistically significant for the PD of the smallest rings i.e., tight central and medium central rings of the SCP and of the tight central ring of the DCP. Also, for the VLD the tight and medium central rings of the SCP were not significantly different in RP patients compared to normal subjects.

The fact that only some central rings showed a sparing of flow impairment could be related to the centripetal course of RP. On the other hand, it is opportune to consider that there is a physiological variation of foveal avascular zone dimension in healthy subjects.

The vessel length density reflects a one-dimensional quantification of the retinal vasculature, (length) and perfusion density a two-dimensional (length and width) measure thus a decrease of PD could be either a reduction of retinal vessel caliber or a decrease in branching patterns. An interesting result of our series is the significant reduction of VLD even in the presence of a normal PD as we found in the tight central ring of the DCP meaning that decrease of branching patterns could be an early biomarker of the disease.

The central macular thickness was also significantly reduced in RP patients compared to controls and there was a significant correlation between CMT and flow impairment.

As previously suggested, we can hypothesize that vascular depletion is a secondary event of inner retinal and outer retinal atrophy due to a reduced metabolic demand. Alternatively, it could be an early event in the disease, which eventually causes ischemia and tissue loss. There is a body of evidence indicating that the reduced ocular blood flow is implicated in RP [5,6,7]. Several histopathologic studies have confirmed that the reduced ocular blood flow is a primary event due to retinal vessel damage. In fact, vessel narrowing and sclerosis, followed by thickening of the blood vessel wall and consequently lumen occlusion, are features of RP [2,3].

A significant reduction of MfERG visual field and microperimetry values has been previously reported in RP patients [5,20,21,22].

In a previous study we found a correlation between macular function and particularly MfERG values and vessel density of parafoveal SCP and DCP.

Alnawaiseh et al. found a significant correlation between flow density of superficial/deep retinal plexuses and visual field parameters such as visual field index and mean deviation in RP patients compared to healthy controls [20].

In the present study, a correlation between functional and anatomical findings was found and in particular between central perfusion density of SCP and CC and retinal sensitivity (at 4° and 8° microperimetry fields) meaning that a reduction of PD implies a decrease of retinal sensitivity.

Our study has several limitations. The series presented here is relatively small, and both the age and the genotype of included patients are not homogeneous. This is due to the rarity of the pathology and also to the limiting inclusion criteria. Another major limitation is that our RP patients were not at the initial stage of the disease, but mostly at the intermediate or late stage, so we were not able to establish if the vascular changes we considered appear earlier or in advanced stage of RP.

In addition we studied only near/mid periphery because of the higher chance to have many shadowing artifacts likely caused by patient’s eyelashes in the peripheral images thus excluding information from the peripheral retina.

In conclusion, we have produced a fully integrated study of retinal and choroidal vessels in RP patients, and found that both the choroid and retinal vessels were modified in these patients compared to normal subjects. Moreover, we demonstrated that the reduction of vessel density and vessel length density correlates with the macular function.

## Figures and Tables

**Table 1:** Clinical and genetic characteristics of patients with RP.

Patient	Gender	Age (Years)	VA	Gene	Mutation/Mutations Identified
*Case 1*	male	*63*	*RE 0.3 logMAR* *LE 0.2 logMAR*	*RPGR*	c.257G > A, p.C86Y
*Case 2*	female	*40*	*RE 0.8 logMAR* *LE 0.7 logMAR*	*NR2E3*	*c.1025T > G, p.V342G*
*Case 3*	male	*59*	*RE 0.4 logMAR*	*PDE6B*	c.1547T > C, p.L516P c.1685G > A, p.G562D
*Case 4*	female	*18*	*RE 0.0 logMAR* *LE 0.1 logMAR*	*EYS*	c.1673G > A, p.W558X c.2811C > A, p.C937X
*Case 5*	female	*51*	*RE 0.0 logMAR* *LE 0.0 logMAR*	*PRPF3*1	c.1060C > T, p.R354X
*Case 6*	male	*41*	*RE 0.2 logMAR* *LE 0.2 logMAR*	*USH2A*	c.688G > A, p.V230M c.2109T > C, p.D703D
*Case 7*	male	*44*	*RE 0.0 logMAR*	*RHO*	c.404G > T, p.R135L
*Case 8*	male	*61*	*RE 0.0 logMAR* *LE 0.1 logMAR*	*CEP290*	c.4040G > A, W1347X c.6964A > T, p.K2322X
*Case 9*	female	*23*	*LE 0.2 logMAR*	*MERTK*	c.2237A > G, p.I631M c.1893C > G, p.K746R
*Case 10*	female	*37*	*RE 0.2 logMAR* *LE 0.1 logMAR*	*USH2A*	c.11946G > A, p.L3982L c.6713A > C, p.E2238A
*Case 11*	male	*54*	*RE 0.7 logMAR*	*AIPL*1	c.834G > A, p.W278X c.834G > A, p.W278X
*Case 12*	female	*66*	*RE 0.4 logMAR* *LE 0.2 logMAR*	*EYS*	c.9082G > T, p.D3028Y c.4350_4356del7, p.K1450KfsX3

**Table 2:** Functional parameters of patients with RP and controls.

Variable	Controls	Cases	Mean Difference (95%CI) *	*p*-Value
**VA (LogMar)**	0.03 ± 0.05	0.24 ± 0.28	−0.27 (−0.43; −0.11)	0.020
**Mf ERG Ring1 (µV)**	139.35 ± 28.27	81.82 ± 36.71	57.43 (47.42; 67.44)	<0.001
**Mf ERG Ring2 (µV)**	63.21 ± 14.19	21.78 ± 9.79	41.43 (35.02; 47.85)	<0.001
**Mf ERG Ring3 (µV)**	33.80 ± 7.36	7.98 ± 3.95	25.82 (22.66; 28.98)	<0.001
**Mf ERG Ring4 (µV)**	22.58 ± 4.78	6.55 ± 5.11	16.02 (13.52; 18.53)	<0.001
**Mf ERG Ring5 (µV)**	16.24 ± 4.06	4.15 ± 3.02	12.09 (10.22; 13.96)	<0.001
**Dark-adapted 0.01 ERG**				
Amplitude (µV)	203.35 ± 22.68	38.94 ± 31.25	164.41 (151.35; 177.48)	<0.001
Latency (ms)	95.70 ± 10.92	88.73 ± 24.07	6.98 (−1.77; 15.72)	0.116
**Dark-adapted 3.0 ERG**				
Amplitude (µV)	210.58 ± 19.62	31.96 ± 29.05	178.62 (166.95; 190.3)	<0.001
Latency (ms)	44.12 ± 6.77	60.11 ± 24.39	−15.99 (−24.23; −7.75)	<0.001
**Light-adapted 3.0 ERG**				
Amplitude (µV)	124.28 ± 16.96	13.26 ± 10.56	111.02 (104.16; 117.92)	<0.001
Latency (ms)	27.54 ± 2.75	38.23 ± 6.15	−10.69 (−12.49; −8.47)	<0.001
**Light-adapted 30 Hz flicker ERG**				
Amplitude (µV)	69.58 ± 10.52	10.22 ± 10.18	59.35 (54.19; 64.52)	<0.001
Latency (ms)	27.97 ± 2.54	34.48 ± 5.81	−6.5 (−8.6; −4.39)	<0.001

* Assessed as controls minus cases values.

**Table 3:** Differences in retinal sensitivity parameters between cases and controls expressed as median and interquartile range (IQR).

Variable	Controls	Cases	*p*-Value ^a^
**4° (dB)**	16.8 (15.0–18.5)	13.6 (9.2–23.8)	0.002
**8° (dB)**	14.2 (13.4–16.8)	10.1 (4.9–20.7)	0.005
**20° (dB)**	11.6 (11.3–14.3)	6.7 (2.8–16.7)	0.004

^a^ Mann-Whitney U Test.

**Table 4:** Perfusion Density values in cases and controls.

Perfusion Density	Controls	Cases	Mean Difference (95%CI) *	*p*-Value
**SCP**				
Medium central ring	34.15 ± 5.19	34.94 ± 6.12	−0.8 (−3.81; 2.22)	0.600
Large central ring	42.09 ± 5.77	37.12 ± 5.41	4.97 (2.07; 7.86)	0.001
Superior ring	46.82 ± 11.41	34.55 ± 11.18	12.26 (6.38; 18.15)	<0.001
Temporal ring	26.75 ± 7.75	21.73 ± 8.04	5.02 (0.97; 9.06)	0.016
Inferior ring	49.46 ± 9.52	36.96 ± 9.38	12.51 (7.58; 17.43)	0.001
Tight central ring	19.81 ± 9.45	23.34 ± 9.31	−5.53 (−9.33; 1.74)	0.121
**DCP**				
Medium central ring	40.32 ± 8.36	28.3 ± 7.68	12.02 (7.87; 16.17)	<0.001
Large central ring	45.64 ± 7.66	30.09 ± 7.25	15.55 (11.68; 19.42)	<0.001
Superior ring	43.13 ± 11.33	31.55 ± 8.67	11.58 (6.48; 16.68)	<0.001
Temporal ring	35.13 ± 7.30	19.63 ± 7.49	15.5 (11.62; 19.37)	<0.001
Inferior ring	48.59 ± 8.44	33.01 ± 6.93	15.58 (11.64; 19.52)	<0.001
Tight central ring	17.16 ± 6.33	17.44 ± 6.54	−0.28 (−3.65; 3.1)	0.870
**CC**				
Medium central ring	84.29 ± 1.86	78.85 ± 3.71	5.43 (3.8; 7.07)	<0.001
Large central ring	84.29 ± 1.80	79.39 ± 3.09	4.9 (3.51; 6.29)	<0.001
Superior ring	83.73 ± 2.26	78.51 ± 3.14	5.22 (3.74; 6.7)	<0.001
Temporal ring	85.84 ± 1.54	77.89 ± 4.90	7.95 (5.88; 10.02)	<0.001
Inferior ring	83.99 ± 1.81	76.41 ± 6.55	7.57 (4.83; 10.32)	<0.001
Tight central ring	83.93 ± 1.78	77.83 ± 4.32	6.09 (4.23; 7.95)	<0.001

* Assessed as controls minus cases values.

**Table 5:** Vessel length density values in cases and controls.

Vessel Length Density	Controls	Cases	Mean Difference (95%CI) *	*p*-Value
**SCP**				
Medium central	19.50 ± 1.43	19.17 ± 1.78	0.33 (1.15; 0.49)	0.423
Large central	19.16 ± 1.01	17.62 ± 1.53	1.54 (2.19; 0.89)	<0.001
Superior	19.83 ± 2.22	16.79 ± 0.98	3.04 (4.01; 2.08)	<0.001
Temporal	20.52 ± 2.00	19.50 ± 2.61	1.03 (0.23; 2.28)	0.106
Inferior	19.66 ± 1.44	16.53 ± 0.72	3.13 (3.77; 2.49)	<0.001
Tight central	20.04 ± 1.53	19.52 ± 2.39	0.52 (1.62; −0.58)	0.351
**DCP**				
Medium central	22.25 ± 2.22	20.80 ± 1.02	1.46 (4.29;1.37)	<0.001
Large central	22.49 ± 2.10	20.88 ± 1.80	1.60 (4.87; −1.66)	0.016
Superior	22.48 ± 2.35	20.15 ± 1.44	2.33 (5.33; 0.66)	<0.001
Temporal	22.93 ± 4.00	20.58 ± 2.38	2.35 (3.97; 0.73)	0.005
Inferior	22.35 ± 4.01	19.63 ± 1.46	2.72 (6.37; 0.92)	0.008
Tight central	17.37 ± 2.92	19.57 ± 1.46	2.2 (3.32; 1.08)	<0.001
**CC**				
Medium central	10.85 ± 0.98	8.66 ± 1.04	2.19 (2.72; 1.67)	<0.001
Large central	10.67 ± 0.78	8.71 ± 1.03	1.95 (2.42; 1.49)	<0.001
Superior	10.35 ± 0.98	8.91 ± 1.35	1.44 (2.04; 0.85)	<0.001
Temporal	10.84 ± 1.35	8.3 ± 1.15	2.54 (3.21; 1.87)	<0.001
Inferior	11.05 ± 1.03	8.89 ± 1.28	2.16 (2.75; 1.57)	<0.001
Tight central	11.1 ± 1.23	8.74 ± 1.09	2.36 (2.98; 1.74)	<0.001

* Assessed as controls minus cases values.

**Table 6:** Pearson’s correlation coefficients among central and peripheral sectors of perfusion density and vessel length density.

	Perfusion Density		Vessel Length Density	
	Controls	Cases	Controls	Cases
**Peripheral sectors SCP**	**Large central SCP**		**Large central SCP**	
**Superior**	0.717 ***	0.010	0.485	0.537 **
**Temporal**	0.804 ***	0.175	0.433 *	0.554 **
**Inferior**	0.857 ***	0.055	0.703 *	0.269
**Peripheral sectors DCP**	**Large central DCP**		**Large central DCP**	
**Superior**	0.713 ***	0.076	0.995 ***	0.301
**Temporal**	0.791 ***	0.181	0.966 ***	0.576 ***
**Inferior**	0.826 ***	0.230	0.993 ***	0.077
**Peripheral sectors CC**	**Large central CCP**		**Large central CCP**	
**Superior**	0.548 **	0.215	0.900 ***	0.162
**Temporal**	0.778 ***	0.251	0.909 ***	0.290
**Inferior**	0.566 **	0.156	0.874 ***	0.075

*** *p* < 0.001; ** *p* < 0.01; * *p* < 0.05.

**Table 7:** Pearson’s correlation coefficients between 4° and 8° microperimetry fields and tight and medium central sectors of perfusion density and vessel length density.

	Microperimetry 4° Ring			Microperimetry 8° Ring	
	Cases	Controls		Cases	Controls
**Perfusion density**			**Perfusion density**		
**Tight central SCP**	0.474 **	−0.036	Medium central SCP	0.309 *	−0.010
**Tight central DCP**	0.115	−0.056	Medium central DCP	0.268	−0.037
**Tight central CC**	0.393 *	−0.357	Medium central CC	0.508 ***	−0.311
**Vessel Length density**			**Vessel Length density**		
**Tight central SCP**	0.245	0.027	Medium central SCP	−0.160	−0.079
**Tight central DCP**	−0.034	0.045	Medium central DCP	−0.438 **	−0.273
**Tight central CC**	−0.337 *	0.354	Medium central CC	−0.498 ***	0.335

*** *p* < 0.001; ** *p* < 0.01; * *p* < 0.05.
